# Tubercular Sinus of Labia Majora: Rare Case Report

**DOI:** 10.1155/2008/817515

**Published:** 2008-02-18

**Authors:** Kela Manoj, Mukherjee Soma, Lunawat Ajay, Agrawal Ashish, Shishodiya Rakesh, R. V. Paliwal

**Affiliations:** ^1^Department of Surgery, Sri Auriboindo Institute of Medical Sciences, Indore, Madhya Pradesh 452001, India; ^2^Department of Obstetrics & Gynaecology, Sri Auriboindo Institute of Medical Sciences, Indore, Madhya Pradesh 452001, India

## Abstract

Tuberculosis of the female external genitalia is unusual and primary infection is rare. We report a 50-year-old female patient admitted to Department to Surgery with swelling over left inguinal area with discharging sinus from labia majora to left inguinal crease which was found to be tubercular sinus on histopathology.

## 1. INTRODUCTION

Tuberculosis (TB) of the vulva and vagina is very rare and it is seen in only 1-2% of genital tract TB. Tuberculosis of cervix accounts for 0.1–0.65% of all cases of TB and 5–24% of genital tract TB [1–8]. Tuberculosis more frequently affects the upper genital tract, namely, the fallopian tubes and endometrium. It usually occurs in women of childbearing age [5, 6, 9].

## 2. CASE REPORT

A 50-year-old female patient without active sexual life admitted to Department to Surgery with a swelling over the left inguinal area with discharging sinus from labia majora to left inguinal crease. She had history of incision and drainage for an abscess at left labia majora 6 months back. She had no history of cough, fever, or abdominal pain. She had not been in close contact with an index case of pulmonary tuberculosis in past year. Antibody tests for HIV and VDRL infection were negative.

Chest and abdominal X-rays were normal. Ultrasonography revealed that the uterus was bulky and endometrial line was not visualized and bilateral adnexae were without a mass or cyst. A full blood count showed leucopenia, and ESR at 2 hours was 55.

**Figure 1:**
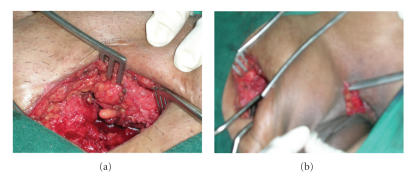


**Figure 2:**
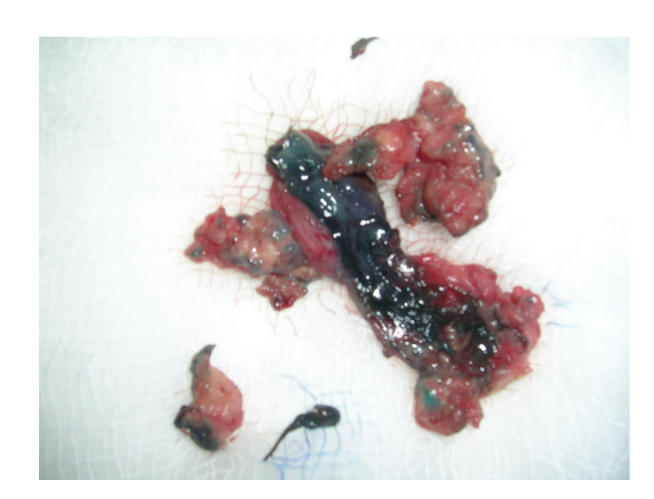


**Figure 3:**
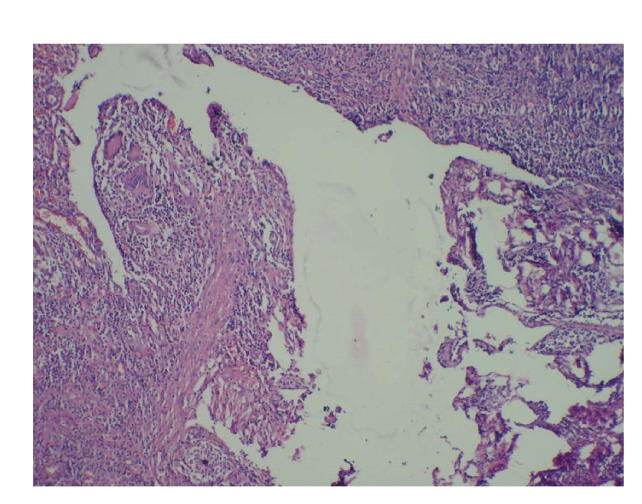


Then patient underwent excision of the sinus tract of labia through suprapubic approach after staining it with methylene blue. A mass of 6×5 cm was excised in the retropubic region (see Figures 1(a), 1(b), and 2). Histopathology report showed sinus tract lined by chronic inflammatory cells, epitheloid cells, and Langhan’s giant cells on microscopic examination suggestive of tuberculous sinus (Figure 3). Antitubreculous quadruple therapy was initiated. Complete healing of the wound, with rapid relief of symptoms, followed 4-week antituberculosis chemotherapy.

## 3. DISCUSSION

Tuberculosis is one of the oldest diseases known to affect humans [10]. Female genital TB is a rare disease in some developing countries, but it is a frequent cause of chronic pelvic inflammatory disease (PID) and infertility in other parts of the world [11]. Symptomatic genital tract TB usually presents with abnormal vaginal bleeding, menstrual irregularities, abdominal pain, and constitutional symptom [5, 6, 9, 12, 13]. Pelvic organs are infected from a primary focus, usually the chest, by haematogenous spread [2, 4, 5, 12, 14]. The cervix is infected as part of this process, by lymphatic spread or by direct extension. The vagina and vulva are rarely involved. The primary lesion is often healed by the time of presentation [5–9, 12–15].

Chowdhury [5] has suggested that sputum, used as a sexual lubricant, may also be a route of transmission. It is uncommon for tuberculosis to involve the vulva and vagina.

The gross appearance may be ulcerative with multiple sinuses, it may be hypertrophic with elephantiasis, or it may be similar to that of carcinoma. There may be hormone dependence of infection [2, 5] given that 80% of cases occur in the reproductive age.

Microscopically, there are caseating granulomatas. These are not diagnostic. The differential diagnosis for granulomatous disease of the cervix includes amoebiasis, schistosomiasis, brucellosis, tularaemia, sarcoidosis, and foreign body reaction. The diagnosis of the cervical and vulvovaginal TB is usually made by histological examination of cervical and vulvovaginal biopsy specimen [3, 9, 14]. Staining for acid-fast bacilli was not found to be very useful in making the diagnosis [16]. The detection of granulomata on cervical cytology specimens [9, 14] has been documented. Isolation of the mycobacterium is the gold standard for diagnosis. One third of cases are culture negative. Therefore, the presence of typical granulomata is sufficient for diagnosis if other causes of granulomatous cervicitis are excluded or primary focus identified. The lesion should respond to 6 months of standard therapy. A lesion on the cervix, vagina, or vulva provides a marker to assess response to therapy. Histological examination of serial biopsy specimens can similarly confirm a therapeutic response.

## References

[1] Carter JR (1990). Unusual presentation of genital tract tuberculosis. *International Journal of Gynecology & Obstetrics*.

[2] Carter J, Peat B, Dalrymple C, Atkinson K (1989). Cervical tuberculosis—case report. *Australian & New Zealand Journal of Obstetrics & Gynaecology*.

[3] Koller AB (1975). Granulomatous lesions of the cervix uteri in black patients. *South African Medical Journal*.

[4] Richards MJ, Angus D (1998). Possible sexual transmission of genitourinary tuberculosis. *The International Journal of Tuberculosis and Lung Disease*.

[5] Chowdhury NN (1996). Overview of tuberculosis of the female genital tracty. *Journal of the Indian Medical Association*.

[6] Kobayashi-Kawata T, Harami K (1978). Tuberculous cervicitis. *Acta Cytologica*.

[7] Chakraborty P, Roy A, Bhattacharya S, Addhya S, Mukherjee S (1995). Tubeculous cervicitis: a clinicopathologyical and bacteriological study. *Journal of the Indian Medical Association*.

[8] Nogales-Ortiz F, Tarancón I, Nogales FF (1979). The pathology of female genital tuberculosis. A 31-year study of 1436 cases. *Obstetrics and Gynecology*.

[9] Shobin D, Sall S, Pellman C (1976). Genitourinary tuberculosis simulating cervical carcinoma. *Journal of Reproductive Medicine*.

[10] Raviglione MC, O'Brien RJ, Fauci AS, Brauwal E, Isselbacher KJ, Wilson JD, Martin JB, Kasper DL (2001). Tuberculosis. *Harrison's Principles of Internal Medicine*.

[11] Martens MG, Rock JA, Thompson JD (1997). Pelvic inflammatory disease. *Telind's Operative Gynecology*.

[12] Sinha R, Gupta D, Tuli N (1997). Genital tract tuberculosis with myometrial involvement. *International Journal of Gynecology and Obstetrics*.

[13] Highman WJ (1972). Cervical smears in tuberculous endometritis. *Acta Cytologica*.

[14] Sutherland AM, Glen ES, MacFarlane JR (1982). Transmission of genito-urinary tuberculosis. *Health Bulletin*.

[15] Bhambhani S, Das DK, Singh V, Luthra UK (1985). Cervical tuberculosis with carcinoma in situ: a cytodiagnosis. *Acta Cytologica*.

[16] Agarwal J, Gupta JK (1993). Female genital tuberculosis—a retrospective clinico-pathologic study of 501 cases. *Indian Journal of Pathology and Microbiology*.

